# Relationship between dietary variety, adequacy, moderation, and balanced diet and cardiovascular risk factors

**DOI:** 10.1186/s40795-022-00514-x

**Published:** 2022-03-08

**Authors:** Mohammad Hossein Sharifi, Payman Izadpanah, Maryam Mohammad Hosseini, Mina Vojoudi

**Affiliations:** 1 Research Center for Traditional Medicine and History of Medicine, Shiraz University of Medical Sciences, Shiraz, Iran; 2 Department of Cardiology, School of Medicine, Shiraz University of Medical Sciences, Shiraz, Iran; 3 School of Nutrition and Food Sciences, Shiraz University of Medical Sciences, Shiraz, Iran

**Keywords:** Myocardial Infarction, Diet Therapy, Heart Disease Risk Factors

## Abstract

**Background:**

The relationship between dietary variety, adequacy, moderation, and balanced diet as diet quality indices and cardiovascular risk factors has not been yet evaluated amongst patients with Mmyocardial Iinfarction (MI).

**Method:**

This cross-sectional study was conducted on 225 males and 93 females with MI who were admitted in two heart hospitals, Shiraz, Iran from November 2019 to April 2020. Dietary intake was assessed using a validated food frequency questionnaire and the Diet Quality Index-International (DQI-I). DQI-I included four subscales, namely variety (20 scores), adequacy (40 scores), moderation (30 scores), and balanced diet (10 scores).

**Results:**

The mean age of the participants was 54 ± 8 years. The mean scores of total DQI-I and variety, adequacy, moderation, and balanced diet subscales were 58 ± 12.2, 12.7 ± 3.8, 28.5 ± 7.0, 9.88 ± 5.6, and 7.8 ± 1.1, respectively. The results showed that waist circumference (WC) was influenced by adequacy (-0.26 ± 0.04) and moderation (-0.28 ± 0.03) subscales, while body mass index (BMI)was only related to the moderation subscale (0.15 ± 0.07).

Additionally, low density lipoprotein (LDL) cholesterol was influenced by variety (-0.18 ± 0.01), adequacy (-0.14 ± 0.02), moderation (-0.2 ± 0.02), and balanced diet (-0.2 ± 0.003) subscales, while total cholesterol was associated with the adequacy subscale (-0.18 ± 0.01). In addition, high density lipoprotein (HDL) cholesterol was related to variety (0.16 ± 0.03), moderation (0.14 ± 0.04), and balanced diet (0.13 ± 0.01) subscales,while triglyceride was only influenced by the variety subscale (-0.15 ± 0.004).

**Conclusion:**

Dietary advice based on variety, adequacy, moderation, and balanced diet could be useful in practice to provide personalized messages to improve the risk factors amongst patients with MI.

## Introduction

Secondary prevention following Myocardial Infarction (MI) has been emphasized in the current guidelines. Since heart diseases play the main role in economic and health burdens of diseases in most countries, diet and lifestyle improvement as a part of secondary prevention has been recommended to improve the risk factors of Coronary Heart Disease (CHD) [1]. Although several studies have evaluated the association between diet quality and CHD, heterogeneous results of a large number of studies have confused healthcare professionals regarding proper recommendations adjusted to risk factors, [2, 3]. Thus, identification of diet quality in patients with MI can provide detailed information to choose proper recommendations in clinical practice based on individual risk factors. Furthermore, limited studies have been conducted on the relationship between Diet Quality Index-International (DQI-I) and its subscales (variety, adequacy, moderation, and balanced diet) and CHD risk factors among patients with MI [4]. The dietary problems recognized by the DQI-I may be valuable in developing strategies to enhance public health prevention program [5]. Hence, the evaluation of the relationship between diet quality indicators and cardiovascular risk factors may help improve secondary prevention programs at both national and local levels.

Contradictory results have also been obtained in large prospective cohort studies examining the association between CHD and diet. Such conflicting results have caused confusion among healthcare providers regarding CHD prevention strategies and advice for MI patients. For example, previous studies indicated that Saturated Fatty Acid (SFA) was related to increased LDL cholesterol level as a CHD risk factor. Therefore, health providers have been recommended to decrease the food sources of SFA. Nonetheless, at least three major review studies showed no relationships between SFA and CHD [6–8]. On the other hand, some studies recommended that the substitution of carbohydrates with SFA could be advantageous in the improvement of lipid profiles [9]. In contrast, Mozaffarian et al. revealed an increase in the CHD risk factors after replacing carbohydrates with SFA in the diet [10]. Therefore, further research is required to determine the association between DQI-I and its subscales (variety, adequacy, moderation, and balanced diet) and CHD, which can provide important information for resolving the aforementioned conflicting results.

The association between diet and CHD is very complex and cannot be attributed to a single food item or nutrient [11, 12]. Besides, indices fore the assessment of the relationship between diet and CHD risk factors have changed over time. Historically, research on the relationship between diet and CHD focused on a single food or nutrient [4]. Accordingly, dietary risk factors for CHD were high-SFA diets and low intake of Polyunsaturated Fatty Acids (PUFA), Monounsaturated Fatty Acids (MUFA), vegetables, fruits, and fiber. More recently, however, the focus has shifted towards the indicators of overall diet quality and dietary patterns to present the nature of diets in the population [12–14]. In this context, diet quality subscales such as variety, adequacy, moderation, and balanced diet that assesses the multidimensional components of the diet, are useful in describing the diet quality and providing the quantitative measures of overall intake relative to dietary guidelines. They can also be used to evaluate the association between diet and risk of chronic diseases [13]. For instance, Carmen Fernandez et al. demonstrated that variety was associated with Body Mass Index (BMI). Thus, dietary variety was recommended for improving BMI. Overall, using diet quality indices, researchers and clinicians have been able to develop a comprehensive approach in diet modification in secondary prevention and to tailor the message based on every risk factor amongst patients with MI. Overall, studies have indicated that CHD risk factors influenced diet. Since limited studies have been done on the relationship between dietary variety, adequacy, moderation, and balanced diet as diet quality indices and cardiovascular risk factors among patients with MI, the present study aims to assess the association between dietary variety, adequacy, moderation, and balanced diet and CHD risk factors to provide insight as to where diets need to be improved and to tailor the message based on every risk factor.

## Method

### Design

This cross-sectional study was conducted on 225 male and 93 female MI patients who were admitted in two specialized heart hospitals from November 2019 to April 2020. The study sample size was calculated based on a previous study [15] and the participants were selected via convenience sampling. The inclusion criteria of the study were not having used any diet therapies in the past 12 months, not having consumed lipid lowering drugs and antioxidants, and not suffering from documented psychological diseases. No exclusion criteria were considered. The participants were required to complete the sociodemographic data questionnaire (gender, age, education level, etc.) and Food Frequency Questionnaire (FFQ) two weeks after the onset of MI.

Anthropometric parameters were assessed at the time of enrollment. Height was measured using a wall-mounted stadiometer to the nearest centimeter while the participants were barefoot. In addition, body weight was measured to the nearest 0.1 kg using a SECA model 770 digital weighing scale (SECA, Hamburg, Germany) while the participants wore light clothes. Then, BMI was calculated by determining the ratio of weight to height squared (kg/m2). Accordingly, BMI > 30 kg/m2 was considered obesity for both males and females [16]. Waist Circumference (WC) was measured using a measuring tape while the patients were in standing position. It was measured at the arrows point between the lower costal margin and the superior iliac crest. In this regard, cut-off points of 102 cm in males and 88 cm in females were considered increased risk for CHD or abdominal obesity [16]. After all, the ideal weight was calculated using Hamwi’s formula, [17],and energy requirement was computed via Harris-Benedict Eq. [18].

### Measurements of food intake and diet quality

Dietary intake was assessed using the FFQ whose validity and reliability have been confirmed previously [19]. The 168-item semi-quantitative FFQ was applied to obtain information on dietary intake over the past year. The nutritional values of the foods reported in the FFQ were analyzed using Nut4 (a computer program for performing a computer-aided nutritional analyze program) followed by the SPSS statistical software, version 22. Afterwards, using the Nut4 output, DQI-I score was computed for each individual [13]. The valid and reliable DQI-I has been used in different studies performed in Iran [20] and around the world [20, 21]. DQI-I gave detailed evaluation of food components and could identify dietary problem areas. This index measured the overall diet quality based on the consumption of food groups and intake of nutrients related to chronic diseases. DQI-I focused on four major aspects, namely variety (0–20 points), adequacy (0–40 points), moderation (0–30 points), and balanced diet (0–10 points). The sum of scores of these four categories could range from 0 to 100, representing the worst and the best possible scores, respectively (Table 2). According to the criteria proposed by Kim et al., total scores below 60% reflected poor-quality diets [13]. It should be noted that all calculations were adjusted for total energy intake, and implausible energy intakes (i.e., < 500 kcal/day or > 4500 kcal/day) were excluded [22].

### Measurements of physical activity

International Physical Activity Questionnaire (IPAQ) is a valid and reliable instrument designed primarily for adults (age range of 15–69 years) [23]. The IPAQ face-to-face interview format was used to assess the participants’ habitual physical activity during seven days before MI. Additionally, energy consumption was calculated based on the second edition of codes and Metabolic Equivalent (MET) values. The IPAQ data were converted to MET scores (MET-min per week) for each type of activity by multiplying the number of minutes dedicated to each activity class by the specific MET score for that activity. Moreover, based on the revised scoring protocol in 2005, physical activity levels were categorized into three levels as follows: high (at least 3000 MET-minutes/week), moderate (at least 600 MET-minutes/week, and low (less than 600 MET-minutes/week).

Measurements of lipid profile: Bloods samples were taken from each patient within 24–48 h after MI. After centrifugation, the sera were separated and serum biomarkers were measured in centralized nutrition laboratories by standard, validated methods. Low Density Lipoprotein-Cholesterol (LDL-C), High Density Lipoprotein-Cholesterol (HDL-C), Triglyceride (TG), and total cholesterol levels were measured through enzymatic colorimetric method using enzymatic kits (Pars Azmoon, Company, Iran). Moreover, abnormal lipoprotein levels were determined based on the national cholesterol education Adult Treatment Panel III (ATP III) diagnostic criteria [16].

### Satistical analysis

The data were expressed as mean ± standard deviation for numeric variables and as frequency or proportion for categorical ones. Kolmogorov–Smirnov test was used for testing the normal distribution of the data. Additionally, distribution of the relevant variables was assessed for outliers or aberrant distributions. Mann–Whitney test, ANOVA, and t-test were used to compare the study groups regarding the variables. Moreover, linear regression model with backward elimination method was employed to assess the associations between the total diet quality, variety, adequacy, moderation, and balanced diet and BMI, WC, LDL cholesterol, HDL cholesterol, total cholesterol, and TG levels. The covariates used in the model for linear regression analysis included blood pressure, total energy intake, physical activity per week, education level, smoking status, and BMI. It should be mentioned that BMI was controlled for all the covariates, except for BMI. All analyses were done using the SPSS statistical software, version 22 and p ≤ 0.05 was considered statistically significant.

## Result

**Fig. 1:**
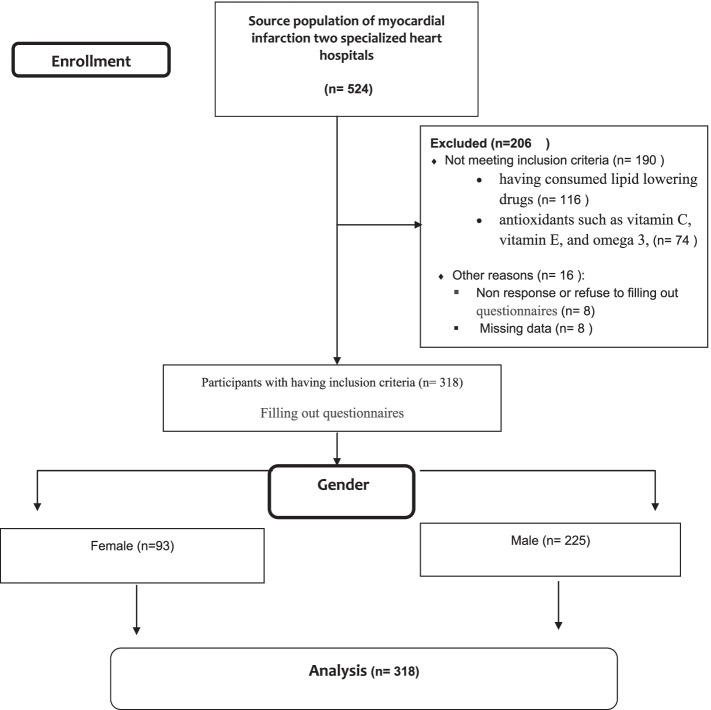
Flow-chart of participants in study

A summary of the study process has been depicted in Fig. 1. The baseline characteristics of the 318 MI patients with the mean age (SD) of 54 ± 8 years have been presented in Table 1. The relationships between the patients’ baseline characteristics and diet quality subscales have also been shown in Table 1. The results revealed significant relationships between diet variety and gender, obesity, LDL cholesterol, total cholesterol, and HDL cholesterol. Dietary adequacy and moderation were also significantly associated with gender, education level, LDL cholesterol, total cholesterol, and HDL cholesterol. Additionally, significant relationships were detected between balanced diet and education level, TG level, and HDL cholesterol. Further details have been provided in Table 1.

**Table 1:** Baseline characteristics of the patients with myocardial infarction and diet quality subscales

Variables			Variety	Adequacy	Moderation	Balanced diet
	*N* = 318	Classified	*N* = 318	*N* = 318	*N* = 318	*N* = 318
**Age** (years) (mean ± SD)	54 ± 8	< 60	13.29 ± 2.87	28.82 ± 6.74	8.65 ± 5.89	7.88 ± 1.32
		40–65	12.63 ± 3.89	29.13 ± 7.12	5.66 ± 0.33	7.80 ± 1.16
		> 65	15.01 ± 2.35	27.50 ± 7.27	4.79 ± 1.28	8.14 ± 1.23
			*P* = 0.063^a^	*P* = 0.698	*P* = 0.607^a^	*P* = 0.546^a^
**Married** (%) (number)	92.7% (295)	Single	10.96 ± 5.12	27.50 ± 5.70	7.8 ± 5.4	7.9 ± 1.2
		Married	12.93 ± 3.64	29.18 ± 7.19	9.9 ± 5.7	7.79 ± 1.12
			*P* = .066^b^	*P* = .170^b^	*P* = .001^c^	*P* = .239^b^
**Gender (Male)** (%) (number)	65.4% (208)	Male	12.2 ± 3.9	28.1 ± 6.5	7.8 ± 5.4	7.9 ± 1.2
		Female	13.8 ± 3.5	30.9 ± 7.7	9.9 ± 5.7	7.7 ± 1. 2
			*P* < .001^c^	*P* = 0.002^b^	*P* = .001^c^	*P* = .317^c^
**Education level** (%) (number)	56.6 (180)	Below high school	12.58 ± 3.61	27.48 ± 6.7	7.65 ± 5.18	7.61 ± 1.11
	18.8 (60)	High school	12.55 ± 3.93	29.62 ± 7.05	8.69 ± 5.7	8.05 ± 1.13
	24.5 (78)	Academic	13.51 ± 7.97	31.1 ± 7.32	10.06 ± 6.11	7.82 ± 1.28
			*P* = 0.190^a^	*P* = 0.001^a^	*P* = 0.015^a^	*P* = 0.011^a^
**Smoker** (%) (number)	8.5% (27)	Yes	13.85 ± 4.26	29.44 ± 6.84	8.89 ± 5.61	8.15 ± 0.95
		No	12.67 ± 3.76	29.01 ± 7.12	8.52 ± 5.65	7.79 ± 1.84
			*P* = 0.123^c^	*P* = 0.758^b^	*P* = 0.743^c^	*P* = 0.125^c^
**Obesity** (BMI (kg/m^2^) > 30) (%) (number)	21.4% (68)	< 30	13.01 ± 3.8	29.30 ± 6.9	8.68 ± 5.61	7.79 ± 1.18
		> 30:	11.90 ± 3.77	28.06 ± 7.73	8.06 ± 5.75	7.91 ± 1.13
			*P* = 0.036^c^	*P* = 0.203^c^	*P* = 0.434^c^	*P* = 0.465^c^
**LDL > 130** mg/dl (%) (number)	31% (98)	< 130	13.24 ± 3.75	30.79 ± 7.10	10.01 ± 5.81	7.79 ± 1.22
		> 130	11.68 ± 3.75	25.01 ± 5.18	> 130: 5.19 ± 3.33	7.63 ± 1.02
			*P* = 0.001^c^	*P* < .001^c^	*P* < .001^c^	*P* = 0.053^c^
**TG > 150** mg/dl (%) (number)	64% (203)	< 150	12.76 ± 3.74	29.52 ± 7.13	9.03 ± 5.68	8.09 ± 1.19
		> 150	11.68 ± 3.75	28.77 ± 7.07	8.28 ± 5.61	7.67 ± 1.13
			*P* = 0.001^c^	*P* = 0.365^c^	*P* = 0.255^c^	*P* = 0.002^c^
**Total cholesterol** > 200 mg/dl (%) (number)	44.7% (142)	< 200	13.57 ± 3.60	30.96 ± 7.32	9.86 ± 5.87	8.09 ± 1.19
		> 200	11.74 ± 3.84	26.61 ± 5.98	6.88 ± 4.86	7.67 ± 1.13
			*P* < .001^c^	*P* < .001^c^	*P* < .001^c^	*P* = 0.002^c^
**HDL for males** ≤ 40 mg/dl (%) (number)	21.7% (69)	< 40	10.74 ± 3.80	27.34 ± 5.50	6.77 ± 4.82	7.62 ± 1.36
		> 40	12.65 ± 3.78	28.29 ± 6.78	6.88 ± 5.59	7.94 ± 1.08
			*P* < .001^c^	*P* = .007^c^	*P* = .001^c^	*P* = 0.037^c^
**HDL for females** ≤ 45 mg/dl (%) (number)	37% (117)	< 45	14.01 ± 2.59	25.42 ± 5.42	5.75 ± 3.72	7.17 ± 1.03
		> 45	13.78 ± 3.62	31.53 ± 7.78	10.50 ± 5.74	7.80 ± 1.20
			*P* = .836^c^	*P* = .003^c^	*P* = .001^c^	*P* = 0.085^c^
**Hypertension history** (blood pressure > 130/90) (%) (number)	17% (54)	Yes	11.80 ± 4.80	28.10 ± 6.14	8.28 ± 5.61	7.68 ± 1.10
		No	12.95 ± 3.58	29.22 ± 7.25	8.60 ± 5.65	7.84 ± 1.83
			*P* = .113^c^	*P* = .255^c^	*P* = .715^c^	*P* = .366^c^

Mean of total energy intake (mean ± SD):2944 ± 449; Percent of carbohydrate intake (gram):58% (435 g); Percent of fat intake (%) (gram): 29% (93.4 g); Percent of protein intake (%) (gram): 13% (90 g)

LDL, low density lipoprotein; HDL, high density lipoprotein; TG, triglyceride. BMI, body mass index

^a^ANOVA Test, ^b^Mann-Whitney Test, ^c^Independent T-test

The mean DQI score of the patients was 58 ± 12.2 out 100. Accordingly, 62.2% of the patients had low total diet quality (total scores below 60). In addition, the mean scores of the variety, adequacy, moderation, and balanced diet subscales of the DQI were 12.7 ± 3.8 (out of 20), 28.5 ± 7.0 (out of 40), 9.88 ± 5.6 (out of 30), and 7.8 ± 1.1 (out of 10), respectively (Table 2).

**Table 2:** The scores of diet quality and its subscales among the patients with myocardial infarction (*n* = 318)

**Component**		**Score**	**Scoring criteria**	
**Variety**				**Mean ± SD**
**Variety (totally 20 scores)**	**Within-group variety for protein source: Total score of 5** **(meat, poultry, fish, dairy products, beans)** **Overall food group variety: Total score of 15 (meat/poultry/fish, dairy products, beans, grains, fruits, vegetables)**	**15**	≥ 1 serving/day from each food group	**9.9 ± 3.3** **(out of 15)**
		12	Any 1 food group missing	
		9	Any 2 food groups missing	
		6	Any 3 food groups missing	
		3	≥ 4 food groups missing	
		0	None from any food groups	
		**5**	≥ 0.5 serving/day from ≥ 3 different sources	**2.8 ± 1.1** **(out of 5)**
		3	≥ 0.5 serving/day from 2 different sources	
		1	≥ 0.5 serving/day from 1 source	
		0	None	
**Total variety score**				**12.7 ± 3.8**
**Adequacy (totally 40 scores)**	**Vegetable group**	**5**	≥ 3–5 servings/day	**1.9 ± 2.4**
		0	0 servings/day	
	**Fruit group**	**5**	≥ 2–4 servings/day	**1.7 ± 2.4**
		0	0 servings/day	
	**Grain group**	**5**	≥ 6–11 servings/day	**5.0 ± 0.0**
		0	0 servings/day	
	**Fiber**	**5**	≥ 20–30 g/day	**1.8 ± 2.4**
		0	0 g/day	
	**Protein**	**5**	≥ 10% of energy	**5.0 ± 0.0**
		0	0% of energy	
	**Iron**	**5**	≥ 100% RDA	**3.9 ± 2.0**
		0	0% RDA	
	**Calcium**	**5**	≥ 100% AI	**4.5 ± 1.1**
		0	0% AI	
	**Vitamin C**	**5**	≥ 100% RDA	**4.7 ± 1.0**
		0	0% RDA	
**Total adequacy score**				**28.5 ± 7.0**
**Moderation(totally 30 scores)**	**Total fat**	**6**	** ≤ 20% of total energy**	**3.2 ± .7**
		3	> 20–30% of total energy	
		0	> 30% of total energy	
	**Saturated fatty acid**	**6**	≤ 7% of total energy	**0.48 ± 1.1**
		3	> 7–10% of total energy	
		0	> 10% of total energy	
	**Cholesterol**	**6**	≤ 300 mg/day	**2.4 ± 2.2**
		3	> 300–400 mg/day	
		0	> 400 mg/day	
	**Sodium**	**6**	≤ 2400 mg/day	**2.0 ± 2.8**
		3	> 2400–3400 mg/day	
		0	> 3400 mg/day	
	**Empty calorie foods**	**6**	≤ 3% of total energy	**1.8 ± 0.9**
		3	> 3–10% of total energy	
		0	> 10% of total energy	
**Total moderation score of diet**				**9.88 ± 5.6**
**Balanced diet(totally 10 scores)**	**Macronutrient ratio (carbohydrates: proteins: fat)**	**6**	55–65:10–15:15–25	**5.7 ± 0.7**
		4	52 to < 55 or > 65 to 68: 9 to < 10 or > 15 to 16: 13 to > 15 or > 25 to 27	
		2	50 to < 52 or > 68 to 70: 8 to < 9 or > 16 to 17: 12 to < 13 or > 27 to 30	
		0	Other	
	**Fatty acid ratio**	**4**	P:S ratio 1–1.5 and M:S ratio 1–1.5	2.1 ± 0.9
		2	P:S 0.8 to < 1 or > 1.5 to 1.7 and M:S 0.8 to < 1 or > 1.5 to 1.7	
		0	Other	
**Total balanced diet score**				**7.8 ± 1.1**
**The patients’ total diet scores (totally 100 scores)**				**58 ± 12.2**

The relationships between the total diet quality and cardiovascular diseases risk factors have been depicted in Table 3. Accordingly, there were significant linear relationships between the total diet quality and BMI (*p* = 0.009), WC (*p* < 0.001), LDL- cholesterol (*p* < 0.001), total cholesterol (*p* = 0.002), and HDL- cholesterol (*p* = 0.001).

**Table 3:** The associations between the total diet quality and risk factors of cardiovascular diseases

Parameter	Linear regression			
	Crud Beta^a^	Coefficient Beta^a^	Standard Error of Coefficients	*P*-value
**BMI (kg/m**^**2**^**)**	0.15	.141	0.144	0.009
**Waist circumference (centimeters)**	-0.43	-0.304	.071	< 0.001
**LDL-cholesterol (mg/dl)**	-0.19	-0.249	.030	< 0.001
**Total cholesterol (mg/dl)**	-0.13	-0.164	.022	0.002
**HDL-cholesterol (mg/dl)**	0.27	0.182	.182	0.001

^a^Adjusted for blood pressure, physical activity per week, education level, smoking status, and energy intake per kilocalories. BMI was controlled for all covariates, except for BMI. Multiple Linear regression model and backward elimination method were used

*LDL* low density lipoprotein, *HDL* high density lipoprotein, *BMI* body mass index

The associations between the diet quality subscales and cardiovascular diseases risk factors have been presented in Table 4. The results demonstrated that each cardiovascular risk factor was influenced by certain subscales of DQI. Accordingly, WC was influenced by the adequacy and moderation subscales of DQI, while BMI was only related to the moderation subscale. LDL-C was influenced by variety, adequacy, moderation, and balanced diet subscales, while total cholesterol was associated with the adequacy subscale. Finally, HDL- cholesterol was related to the variety, moderation, and balanced diet subscales,while and TG was only influenced by the variety subscale.

**Table 4:** The associations between the diet quality subscales and cardiovascular diseases risk factors

Parameters	Variety			Adequacy			Moderation			Balanced diet		
	Coefficient Beta	*P*-value	SEC	Coefficient Beta	*P*-value	SEC	Coefficient Beta	*P*-value	SEC	Coefficient Beta	*P*-value	SEC
**BMI** (kg/m^2^)	-	-	-	0.110	0.064	0.090	0.150	0.008	0.070	-	-	
**Waist circumference (centimeters)**	-	-	-	-0.260	< 0.001	0.040	-0.280	< 0.001	0.03	-	-	-
**LDL-cholesterol (mg/dl)**	-0.22	< 0.001	0.010	-0.160	0.005	0.020	-0.21	< 0.001	0.01	- 0.210	0.001	0.003
**Total cholesterol (mg/dl)**	-	-	-	- 0.170	0.003	0.010	-0.100	0.075	0.010	0.120	0.052	0.002
**Triglyceride (mg/dl)**	0.16	0.008	0.004	-	-	-	-	-	-	-	-	-
**HDL-cholesterol (mg/dl)**	0.19	0.001	.0240	0.110	0.036	0.040	0.150	0.005	0.030	0.140	0.016	0.010

Adjusted for blood pressure, total energy intake, physical activity per week, education level, smoking status, and BMI. BMI was controlled for all covariates, except for BMI. Multiple linear regression model and backward elimination method were used

*SEC* standard error of coefficients, *LDL* low density lipoprotein, *HDL* high density lipoprotein, *BMI* body mass index

## Discussion

The association between diet and CHD is very complex and recently, the focus has shifted towards the indicators of overall diet quality and dietary patterns to present the nature of diets. The present study findings showed that LDL was influenced by variety, adequacy, moderation, and balanced diet subscales, while total cholesterol was associated with the adequacy subscale. In addition, HDL cholesterol was related to variety, moderation, and balanced diet subscales and triglyceride was only influenced by the variety subscale.

One of the important findings of the current study was that 62.8% of the participants had low total quality scores, and 850 kcal extra calories were averagely consumed per day. Based on the criteria proposed by Kim et al., the mean score of DQI was low in the current study. Consistently, the prior studies indicated that the mean score of DQI was 59.8 ± 11.7 in patients with acute coronary syndrome [24], 68.9 ± 8.2 in patients with diabetes [21], 59.1 ± 0.14 in the normal population in the United States, and 60.5 ± 0.11 in the normal population in China [13]. However, considerably different results were obtained regarding the moderation subscale (Table 2). The mean score of the moderation subscale was 9.88 ± 5.6 in the current study, but 14.3 ± 0.08 in the normal population in the United States and 18.6 ± 0.10 in the normal population in China.

According to Table 2, the results related to the adequacy subscale indicated that intake of fruits, vegetables, and fiber was lower than 50% of the required level. Previous studies indicated that increased consumption of fruits, vegetables, grains, fiber, and protein was positively associated with decreased LDL-C and total cholesterol levels [25]. Besides, the results related to the moderation subscale indicated that the lowest scores were related to SFA, empty calorie foods, and sodium intake. This might be relatively due to the 850 kcal extra calories consumed per day. The results also showed that 36% of the participants had favorable LDL-C levels, which might be due to the moderately high carbohydrate diets (57% of energy). Overall, low carbohydrate diets were not shown to be a health benefit after MI [26].

Increased BMI and WC had deleterious effects on the risk of CHD in both sexes across diverse populations. [27, 28] The results of the present study indicated that WC and BMI were significantly related to the diet quality (Table 3). WC could play a more efficient role in the elucidation of obesity-related health risks [29]. This was partially in agreement with the results obtained by Dara J Ford, reporting that diet quality was significantly higher among overweight individuals, but lower among obese ones [30]. Yet, this was partly on the contrary to the results of the study by Kala Sundararajan, which demonstrated that diet quality was associated with lower BMI only in obese individuals [31].

The present study results revealed a significant association between BMI and the moderation subscale (Table 4). On the other hand, WC showed significant associations with the moderation and adequacy subscales. This might be due to the effects of the components of the adequacy subscale (fruits, vegetables, grain group, fiber, and protein) on decreasing the visceral fat. The previous studies indicated that increased consumption of fruits, vegetables, grain group, fiber, and protein was negatively associated with BMI and WC [32, 33]. Hence, it seems that increasing the scores of the moderation, adequacy, and balanced diet subscales could be helpful in the improvement of WC as a CHD risk factor. However, the current study results revealed no significant relationship between BMI and WC, and the variety subscale. In contrast, Fernandez et al. showed that high dietary variety was associated with higher BMI among children [14]. This discrepancy might be attributed to the difference in the populations under investigation. In addition, a previous study revealed an association between lipid profiles and diet quality measured by the healthy eating index [34]. The results of the current study demonstrated that 31%, 44.7%, and 64% of the participants had abnormal LDL-C, cholesterol, and TG levels, respectively.

## Conclusion

This study aimed to determine the relationships between dietary variety, adequacy, moderation, and balanced diet as diet quality indices and cardiovascular risk factors among patients with MI. The findings revealed that dietary quality was relatively poor amongst the patients, and all the cardiovascular risk factors were related to dietary quality subscales such as variety, adequacy, moderation, and blanched diet indices. To promote secondary prevention in patients with MI, professional health workers need more detailed information about diet quality to improve the risk factors in clinical practice.Thus, as a secondary prevention approach, improvement of the variation, moderation, and adequacy subscales should be taken into consideration in MI patients’ training and consultation. Overall, it seems that diet recommendation based on diet quality can be helpful in practice to improve the CHD risk factors.

## Data Availability

Data is available from the corresponding author on request**,** mhsharifi1350@gmail.com Data is available if needed.
